# CEOs’ Financial Background and Non-financial Enterprises’ Shadow Banking Business

**DOI:** 10.3389/fpsyg.2022.903637

**Published:** 2022-06-28

**Authors:** Chen Yang, Weitao Shen

**Affiliations:** School of Management, Xiamen University, Xiamen, China

**Keywords:** financial background, shadow banking, overconfidence, entity investment, off real to virtual

## Abstract

In recent years, the “financial-like” behavior of non-financial enterprises has contributed to the “off real to virtual,” which has seriously restricted the virtuous cycle of finance and economy. This study selects non-financial enterprises listed on Chinese A-shares from 2008 to 2019 as the research sample, and empirically analyzes the impact of CEOs’ financial background (FB) on the shadow banking business of non-financial enterprises and its mechanism. The results show that: (1) CEOs’ FB has a positive effect on shadow banking business of non-financial enterprises; among which, the positive effect generated by non-banking FB is stronger. The conclusions still hold after robustness tests by replacing the measurement of variables, controlling for other shocks, changing the parameter estimation method, and considering the endogeneity problem. (2) The mechanism analysis reveals the positive effect mainly by reducing the level of entity investment by enterprises. (3) The heterogeneity analysis finds that, on the one hand, with respect to the internal micro characteristics of enterprises, the positive effect is more significant in state-owned enterprises, non-manufacturing enterprises, and non-growth stage enterprises. On the other hand, with respect to the external macro environment, the positive effect is more significant in periods of easy monetary policy, in industries with a higher competition or in regions with a better institutional environment. This study reveals the intrinsic mechanism of CEOs’ FB and non-financial enterprises’ shadow banking business, enriches the study of the influencing factors of non-financial enterprises’ shadow banking business, and provides micro-level empirical support to alleviate the “off real to virtual” of the economy.

## Introduction

At present, non-financial enterprises in China are increasingly turning their resources toward virtual assets due to the increasing uncertainty in global economic policy, especially due to the huge impact of COVID-19. Increasingly, non-financial enterprises are devoting their resources to shadow banking business, including entrusted loans at higher costs, exacerbating corporate governance problems. Shadow banking, which plays the role of “bank-like,” is a variety of financial intermediary businesses outside the conventional banking system (Acharya et al., 2013). Shadow banking usually relies on non-bank financial institutions as carriers to convert risk factors such as credit, liquidity, and maturity of financial assets (Guo and Xia, 2014). As it turns out, shadow banking has existed in China for some time. It has been part of China’s financial system since the 1980s (Allen and Gu, 2020; Zhu, 2021). By the end of 2019, the scale of broad shadow banking in China was RMB 84.80 trillion, while that in the narrow sense was RMB 39.14 trillion.^1^ There is a hidden debt network and guarantee system in shadow banking. The continued convergence of systemic risks in the financial market may accelerate the spread of financial risks. Suppose there is a substantial impairment of financial assets or the bursting of bubbles. In that case, enterprises will fall into a serious financial dilemma, increasing in non-performing loans and corporate defaults, and destabilizing the stability of the traditional financial system and the real economy (Liang, 2016; Jiang and Fang, 2021). Governments have become increasingly concerned with shadow banking governance as one of the most important ways to prevent systemic financial risks.

**FIGURE 1:**
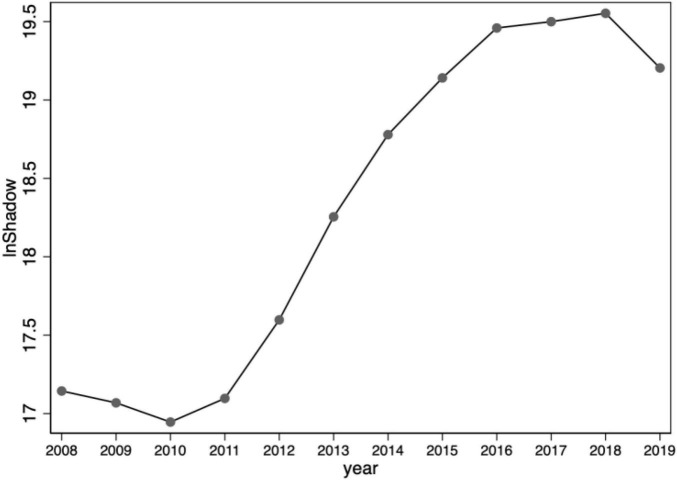
Trends in shadow banking business of non-financial enterprises, 2008–2019.

Shadow banking has attracted the attention of practical and theoretical circles due to its rapid expansion. The majority of existing studies on shadow banking have focused on the off-balance sheet business of commercial banks, the channel business of banks, securities and insurance, and various quasi-financial institutions, etc. There has been little attention paid to the lending behavior of the enterprise sector in emerging markets and transition economies (Du J. L. et al., 2017; Rapih, 2021). The re-lending of the enterprise sector is usually found in emerging markets and transitional economies where financial development is low and the financial system has not yet been developed (Wang et al., 2015; Han et al., 2019). Along with the growing gap between financial and real yields, the corporate sector has a growing tendency to engage in shadow banking business. Figure 1 shows the trend of shadow banking business of sample enterprises from 2008 to 2019, which is in line with the trend of shadow banking development in China. Since the financial crisis in 2008, the growth rate of shadow banking slowed down; from 2011 to 2016, shadow banking entered a rapid growth phase; since the beginning of 2017, the financial management department has focused on rectifying the chaos of shadow banking, and the scale of shadow banking dropped significantly from the historical high. The growth rate declined significantly after 2018. Although there have been more studies on the shadow banking system and financialization of enterprises, there are still relatively few studies on the shadow banking business of non-financial enterprises. The academic has explored shadow banking business of non-financial enterprises based on different research perspectives. For example, identification of shadow banking activities (Wang et al., 2015), social welfare loss (Liu et al., 2014), business and operating risks (Li and Han, 2019), systemic risk (Allen et al., 2019), stock price crash risk (Si et al., 2021), business performance (Han et al., 2019), etc.

To discuss the long-term mechanism of shadow banking governance, we need to focus on its root. According to the Upper Echelons Theory, executives’ different traits and experiences impact business management and strategic decisions (Dearborn and Simon, 1958; Hambrick, 2007; Mun et al., 2020). In addition, the human capital theory suggests that the knowledge and competencies possessed by managers can be significant factors of organizational performance (Amit and Shoemaker, 1993; Coff, 2002). Thus, as the maker and executor of corporate decisions, the CEO’s characteristics will play a crucial role in the decision-making of enterprises shadow banking business. Thus, it would be invaluable to examine the influence factors of shadow banking business in non-financial enterprises by analyzing the background characteristics of CEOs. However, little literature has focused on the influencing factors of non-financial enterprises engaged in shadow banking business, especially the impact of the background characteristics of those managers who play a leading role in shadow banking business of non-financial enterprises. Based on the research on the background characteristics of managers, few studies directly explore the relationship between CEOs’ financial backgrounds (FBs) and their classification and the shadow banking business of non-financial enterprises. The imprinting theory holds that executives’ growth, learning, and work experiences during sensitive periods may produce a psychological imprint that has a lasting cognitive and competence effect on their careers (Mathias et al., 2015). Given the unique operation mode and the highly stressful environment of the financial industry, CEOs’ time in this industry becomes a “sensitive period” (Du et al., 2019). Therefore, the experience of working in the financial industry leaves CEOs with a deeper memory and habit, which will have a significant impact on their career behavior. Our study assumed that the CEO had FBs if they had worked in regulatory agencies, policy banks, or other financial institutions. Then, do the FBs of the CEO and their classification affect the shadow banking business of non-financial enterprises? And what will be the impact? How do CEOs with FBs affect the shadow banking business of non-financial enterprises? And what is the mechanism of the influence between the two? China is currently undergoing a critical period of economic transformation. Thinking on the above issues is helpful to clarify the influencing factors of shadow banking business of non-financial enterprises from the perspective of managerial behavior, restrain the tendency of shadow banking business of non-financial enterprises from the root, promote China’s future economic system reform, and achieve high-quality economic development.

Our study makes the following potential contribution. First, it explores the relationship between “CEOs’ FBs and shadow banking business of non-financial enterprises,” which extends the study of the influencing factors of non-financial enterprises’ shadow banking business, and enriches the study of the impact of CEOs’ FBs and their classification on the real economy. In this way, the factors that influence shadow banking business of non-financial enterprises in emerging markets can be clarified. This can also provide a reference for better regulating the shadow banking business of non-financial enterprises and promoting high-quality economic and financial development. Second, we examined the heterogeneity of the impact from the internal micro characteristics and the external macro environment of enterprises. We are committed to analyzing whether the impact is different for non-financial enterprises with different equity, industry, and life cycles. Moreover, we are committed to analyzing whether the impact differs for non-financial enterprises in monetary policy periods, industry competition levels, and institutional environments. Third, using the mediating effect model constructed by Wen and Ye (2014), we identified the path between CEOs’ FBs and non-financial enterprises’ shadow banking business based on “overconfidence and the entity investment level,” and revealed the influencing mechanisms and deeper logical relationship.

The main structure is organized as follows: section “Literature Review and Hypothesis Development” reviews the literature and presents the development of the hypothesis, section “Research Design” describes the research design, section “Empirical Results and Analysis” presents the empirical results and analysis, section “Mechanism Analysis” performs mechanism analysis, section “Extended Analysis” conducts the extended analysis, and section “Conclusion and Implications” discusses the conclusion and implications.

## Literature Review and Hypothesis Development

Shadow banking in China is dominated by banks. It is primarily made up of commercial banks and non-banking institutions with close ties to banks. Its uniqueness is primarily manifested in financial products, bank-trust cooperation, private finance, etc. The emergence of shadow banking evades the need for various loan restrictions imposed by the central government on banks. It is a disguised loan and the shadow of banks. The participants of shadow banking are usually non-bank financial institutions that stray outside the boundaries of financial regulation (Acharya et al., 2013). In addition, China’s financial repression, underdeveloped financial capital markets, and the lack of supervision of shadow financial activities have led non-financial enterprises to invest in the credit market and engage in the shadow banking business with high risk and high interest rates. Non-financial enterprises have become participants in shadow banking activities (Liu et al., 2014; Wang et al., 2015). The upper echelon theory suggests that managers will have a significant impact on the strategic behavior of the organization, and that the personal characteristics of managers will have an impact on the organization’s results (Hambrick and Mason, 1984). In previous studies, managerial characteristics have been demonstrated to have a significant impact on enterprises’ financial decisions (Gore et al., 2011; Malmendier et al., 2011). Managers with FBs have a strong financial consulting function, which will affect enterprises’ financial decisions (Gore et al., 2011; Malmendier et al., 2011). As an important financial decision, non-financial enterprises engaged in shadow banking business will inevitably be affected by the FBs of managers.

On the one hand, non-financial enterprises evade regulation through non-traditional financing channels, convert maturity and liquidity, and create many non-monetary financial assets (Gennaioli et al., 2013). Shadow banking business of non-financial enterprises will inevitably exacerbate the trend of industrial hollowing out, and strengthen the cross-contagion effect between enterprise sectors and between enterprise sectors and financial markets. The existence of shadow banking will increase in systemic risk (Liang, 2016; Fan and Pan, 2020; Pan and Fan, 2020). CEOs who have worked in financial institutions are more financially sophisticated (Custodio and Metzger, 2014) and can endure greater investment risks, navigate more complex investment environments, and manage more diverse investment projects. In addition, CEOs with FBs may perceive and manage financial investment risks, process and react to financial-related information, and identify and select financial investment opportunities more effectively than CEOs without FBs (Du et al., 2019; Cheng and Wu, 2022). Compared with CEOs without FBs, CEOs with FBs are more likely to be better at identifying, coping with, and taking risks, and more daring in participating in shadow banking business, and, thus, their enterprises have a higher degree of shadow banking.

On the other hand, non-financial enterprises have an extremely good reason to get involved with and expand shadow banking businesses because of financing constraints (Chen et al., 2001). The credit regulatory policies adopted in China seriously restrict the ability of enterprises to obtain capital in the existing capital market, and restrict commercial banks from providing credit to certain types of enterprises (Song et al., 2011). CEOs with FBs have richer financial resources than CEOs without FBs. According to the theory of social capital, they can use their financial “circles” and “guanxi” to provide invisible guarantees for their firms, build access to financial resources (Guner et al., 2008), and integrate more funds from capital markets and financial intermediaries. Signal theory suggests that the FB of the CEO might convey credit advantage signals upstream and downstream in the supply chain (Zhang and Wiersema, 2009; Bernile et al., 2018). Companies with CEOs with FBs can also be suitable lenders upstream and downstream of the supply chain, which participates in the shadow credit market by acting as a bank that provides funding to firms with financial difficulties.

In addition, managers will focus more on achieving higher short-term performance to make profits. Shadow banking business may bring more short-term profits, conducive to managers dealing with short-term assessment pressure (Sen and Dasgupta, 2018). Deng et al. (2021) studied that compensation deferral policy induced CEOs to engage more in shadow banking business to avoid regulating balance sheet risks and improve performance. CEOs with FBs possess financial expertise and a background in the financial industry, which gives them an advantage in financial investment (Francis et al., 2015). Non-financial enterprises may find that shadow banking business provides a higher return on investment than real economy investments when faced with a low return on real investment and a lack of real investment opportunities (Tori and Onaran, 2018). Due to the pressures of performance assessment and self-worth realization, CEOs with FBs are more inclined to utilize their advantage and allocate the firm’s resources to financial investments while reducing industrial investments (Liu et al., 2020). If properly positioned, this type of investment can generate excellent short-term profits for the organization.

Therefore, the enterprises whose CEOs have FBs have a higher degree of shadow banking business. Accordingly, we propose the following hypothesis:

**H1:** Enterprises with CEOs with FBs are more likely to engage in the shadow banking business, that is, the larger their shadow banking business is.

The upper echelons theory states that individuals or teams can only gather limited information due to objective realities. Even if they work with the same information, different managers will make different judgments based on their heterogeneous experiences, values, and cognition, and then make differentiated strategic decisions (Hambrick and Mason, 1984). However, there are differences between different financial institutions regarding their business scope, rules, risk management, resource attributes, and other characteristics, which create a differentiated environment (Du et al., 2019; Cheng and Wu, 2022). Therefore, CEOs from different financial institutions have differing characteristics, which will affect their decision-making processes.

CEOs with previous experience in non-bank financial institutions exhibit a risk-taking style that leads to their engagement in shadow banking activities. Upper echelons theory states that managers’ experiences influence their decisions and preferences (Hambrick and Mason, 1984). Generally, traditional banks, which are primarily engaged in lending and settlement, have stable sources of funds. They have a relatively homogeneous business and low risks. Non-bank financial institutions have more types of business, a more complex working environment and higher risks (Du et al., 2019). As most of the collective investment in shadow banking business is financial innovation tools, such as structuring and asset securitization with high risks, it is generally faced with run-and-fire sale losses (Hanson et al., 2015). Moreover, the maturity mismatch characteristic of structured investment, which is unique to shadow banking, will trigger its inherent instability and aggravate the evolution of risks (Hanson et al., 2015). CEOs who worked for non-bank financial institutions had a higher risk appetite, resulting in a riskier investment strategy (Hertwig et al., 2004; Du et al., 2019). Therefore, compared with CEOs with banking backgrounds, CEOs with non-banking FBs are considered to have higher risk preferences and are more likely to engage in shadow banking business. Our hypothesis is, thus, as follows:

**H2a:** Enterprises with CEOs with non-banking FBs are more likely to engage in the shadow banking business, that is, the larger their shadow banking business is.

CEOs’ experience in banks enables them to exploit their advantages in resource acquisition and financial skills, allowing non-financial enterprises to become involved in shadow banking business. With the “vacancy” in formal institutions, we can see the role of informal institutions, such as social capital and interpersonal relationships, in the transition of the economy (Peng and Luo, 2000). In China, the capital market is still in the initial stages of development, and the primary source of financing is the credit granted by banks. As a result of banks’ reluctance to lend, enterprises have difficulty obtaining financing and are more likely to suffer financial deterioration (Baum et al., 2009; Zhu, 2021). CEOs with banking backgrounds are ideally positioned to develop new capital flow channels for enterprises because of the financial resources during their tenure in banks. CEOs with bank backgrounds are likely to have enhanced resource acquisition skills, and their organizations have access to various sources of funds to finance enterprises that may be suffering from financing restrictions (Allen et al., 2019). Furthermore, managers may prefer areas they are familiar with when faced with multiple investment options. Shadow banking is a form of financial innovation that stimulates lending behaviors for enterprises by expanding existing investment and generating new investments (Simsek, 2013). In shadow banking, the financial products carried out by banks account for a significant portion. There are various financial products, such as credit loans, beneficial rights of trust, entrusted loans, specific asset income, and asset pools. CEOs who have worked in banks are more familiar with the process and scheme of shadow banking business, and can provide guidance and suggestions for shadow banking business of non-financial enterprises (Francis et al., 2015). Accordingly, we propose the following hypothesis:

**H2b:** Enterprises with CEOs with non-banking FBs are more likely to engage in the shadow banking business, that is, the smaller their shadow banking business is.

## Research Design

### Sample and Data

This study selects Chinese A-share-listed companies from 2008 to 2019 as the research sample. These companies were screened as follows: (1) excluded listed companies in the financial and insurance sectors, and listed companies in the real estate sector; (2) excluded ST and *ST companies; (3) excluded financial-listed companies with missing data; (4) we performed all continuous variables in the model with 1 and 99% tailing to mitigate the influence of an extreme value on the research findings; and (5) all standard errors of regression results are adjusted by firm-level clustering to control for potential autocorrelation problems. We obtained the data for this study from the China Stock Market and Accounting Research (CSMAR) database. In total, we obtained 27,782 firm-years observations from 3,504 companies.

### Main Variables

#### Explained Variables

The size of shadow banking business of non-financial enterprises (*lnShadow*). Referring to Jiang et al. (2010) and Han et al. (2019), we measured the size of shadow banking business by taking the logarithm of the sum of entrusted loans, entrusted finance, and private lending. (i) Entrusted loans. To estimate entrusted loans, we selected other current assets, non-current assets with maturity within 1 year, and other non-current assets from the “Balance Sheet” of the CSMAR database company research series – Financial Statements Section. (ii) Entrusted finance. We selected entrusted finance from the “Listed Companies’ Entrusted Finance Sheet” in the CSMAR database company research series – Foreign Investment Section. (iii) Private lending. We selected other receivables as a proxy variable for private lending from the “Balance Sheet” in the CSMAR database company research series – Financial Statements Section.

#### Explanatory Variable

CEO’s FB (*CEO_Fin*). Referring to Du et al. (2017), when the CEO has worked in policy banks, commercial banks, investment banks, financial regulators, fund management companies, insurance companies, exchanges, securities companies, securities registration and settlement companies, futures companies, trust companies, investment management companies, and other financial institutions, the value is 1. Otherwise, the value is 0. Moreover, this study examines the factors affecting the shadow banking business of non-financial enterprises by categorizing CEOs’ FBs into bank-only backgrounds (*Bank_Fin*) and non-bank backgrounds (*NBank_Fin*). Namely, (i) CEO’s banking background (*Bank_Fin*). It takes the value of 1 when the CEO has only worked in a banking-type financial institution; otherwise, it is 0. (ii) CEO’s non-banking FB (*NBank_Fin*). The value is 1 when the CEO has only worked in non-bank financial institutions; otherwise, it is 0.

#### Control Variables

Referring to Han et al. (2019) and Deng et al. (2021), and other studies on shadow banking business of non-financial enterprises, we introduced the following control variables that may affect shadow banking business of non-financial enterprises: enterprise size (*InSize*), enterprise age (*Age*), nature of equity (*State*), profitability (*ROA*), enterprise growth (*TobinQ*), financial leverage (*Lev*), current assets ratio (*Liquidity*), CEO duality (*Dual*), largest ownership (*LShare*), and the proportion of independent directors (*Indep*). In addition, we controlled for year-and-industry-fixed effects in the regression analysis results to mitigate potential endogeneity issues. The specific variables are defined and constructed as shown in Table 1.

**TABLE 1:** Variable definition and description.

Type	Variables	Symbol	Description of variables
Explained variables	The size of shadow banking business banking	*lnShadow*	*ln* (entrusted loan + entrusted finance + private lending)
Explanatory variables	CEO’s financial background	*CEO_Fin*	If the CEO has a financial background, the value is 1; otherwise, the value is 0
	CEO’s banking background	*Bank_Fin*	If the CEO has worked only in banking financial institutions, the value is 1; otherwise, the value is 0
	CEO’s non-banking financial background	*NBank_Fin*	If the CEO has worked only in non-banking financial institutions, the value is 1; otherwise, the value is 0
Control variables	Enterprise size	*lnSize*	*ln*(total assets)
	Enterprise age	*Age*	Enterprise establishment year
	Nature of equity	*State*	If a firm is state-owned enterprise, the value is 1; otherwise, the value is 0
	Profitability	*ROA*	The ratio of net profit to total assets
	Enterprise growth	*TobinQ*	(Stock market value + total debts)/total assets
	Financial leverage	*Lev*	The ratio of total debt to total assets
	Current assets ratio	*Liquidity*	The ratio of total current assets to total assets
	CEO duality	*Dual*	If the chairman is also the CEO, the value is 1; otherwise, the value is 0
	Largest ownership	*Lshare*	The shareholding ratio of the largest shareholder of the enterprise
	The proportion of independent directors	*Indep*	The ratio of the number of independent directors to the total number of directors

### Baseline Regression Model

We studied the impact of CEOs’ FB on shadow banking business of non-financial enterprises. According to the research hypothesis, we constructed an econometric model of shadow banking business of non-financial enterprises as the explained variable; and CEOs’ FB, CEOs’ banking background and CEOs’ non-bank FB as explanatory variables.


(1) $$l\,n\,S\,h\,a\,d\,o\,w_{i,t}=\alpha_{0}+\alpha_{1}\,C\,E\,O\,\_\,F\,i\,n_{i,t}+\alpha \,C\,o\,n\,t\,r\,o\,l_{i,t}+I\,n\,d\,u\,s\,t\,r\,y+Y\,e\,a\,r+\varepsilon_{i,t}$$

(2) $$l\,n\,S\,h\,a\,d\,o\,w_{i,t}=\beta_{0}+\beta_{1}\,B\,a\,n\,k\,\_\,F\,i\,n_{i,t}+\beta_{2}\,N\,B\,a\,n\,k\,\_\,F\,i\,n_{i,t}+\beta \,C\,o\,n\,t\,r\,o\,l_{i,t}+I\,n\,d\,u\,s\,t\,r\,y+Y\,e\,a\,r+\varepsilon_{i,t}$$

where *i* represents the listed company and *t* represents the time; *lnShadow* represents the scale of shadow banking business; *CEO_Fin* represents whether the CEO has a FB; *Bank_Fin* indicates whether the CEO has only a banking background; *NBank_Fin* represents whether the CEO has only a non-banking background; *Control* is the control variable. On this basis, we also incorporated industry-and-time-fixed effect. In Model (1), we analyzed the impact of CEOs’ FB on shadow banking business of non-financial enterprises. According to our research hypothesis, we expected α_1_ > 0; In Model (2), we describe the impact of CEOs’ banking background and CEOs’ non-banking background on shadow banking business of non-financial enterprises. If β_1_ < β_2_, H2a is supported; otherwise, H2b is supported.

## Empirical Results and Analysis

### Descriptive Statistics

Table 2 reports the results of descriptive statistics for the main variables. The mean of the shadow banking scale (*lnShadow*) is 18.5600, and the standard deviation is 2.1190, with a gradually increasing trend from 25 to 90%, which is in line with the current development in China. The mean of *CEO_Fin* is 0.0451, indicating that CEOs with FBs accounted for only 4.51%. Among them, the mean of *Bank_Fin* is 0.0104, and that of *NBank_Fin* is 0.0348, indicating that the proportion of CEOs with non-banking FBs is larger.^2^ The mean value and standard deviation of the control variables are roughly in accordance with the statistical results of other studies.

**TABLE 2:** Descriptive statistical results of major variables.

Variables	*N*	Mean	SD	P25	P50	P75	P90
*lnShadow*	27,782	18.5583	2.1190	17.1046	18.5679	20.0086	21.3429
*CEO_Fin*	27,782	0.0451	0.2076	0.0000	0.0000	0.0000	0.0000
*Bank_Fin*	27,782	0.0104	0.1013	0.0000	0.0000	0.0000	0.0000
*NBank_Fin*	27,782	0.0348	0.1832	0.0000	0.0000	0.0000	0.0000
*lnSize*	27,782	21.9705	1.2604	21.0578	21.8056	22.6877	23.6664
*Age*	27,782	16.0565	5.7274	12.0000	16.0000	20.0000	24.0000
*State*	27,782	0.3796	0.4853	0.0000	0.0000	1.0000	1.0000
*ROA*	27,782	0.0356	0.0752	0.0140	0.0380	0.0681	0.1015
*TobinQ*	27,782	2.6863	2.0527	1.4236	2.0405	3.1847	4.9339
*Lev*	27,782	0.4254	0.2177	0.2534	0.4133	0.5814	0.7132
*Liquidity*	27,782	0.5609	0.2061	0.4183	0.5774	0.7199	0.8242
*Dual*	27,782	0.2691	0.4435	0.0000	0.0000	1.0000	1.0000
*Lshare*	27,782	0.3473	0.1475	0.2315	0.3282	0.4482	0.5532
*Indep*	27,782	0.3729	0.0528	0.3333	0.3333	0.4286	0.4286

### Analysis of Baseline Model Estimation Results

First, CEOs’ FB and shadow banking business of non-financial enterprises. To examine the potential impact of CEOs’ FB on the shadow banking business of non-financial enterprises, we estimated the parameters of the model shown in Equation 1. The regression results are shown in Column (1) of Table 3. The coefficient of *CEO_Fin* is significantly positive at the 5% level, indicating that the CEOs’ FB is positively correlated with the shadow banking business of non-financial enterprises. H1 is verified.

**TABLE 3:** CEOs’ FB and its categories and shadow banking business of non-financial enterprises.

	(1) *lnShadow*	(2) *lnShadow*
*CEO_Fin*	0.1542** (2.5416)	
*Bank_Fin*		0.1118 (0.8755)
*NBank_Fin*		0.1667** (2.4093)
*lnSize*	1.0900*** (59.3066)	1.0899*** (59.2811)
*Age*	0.0028 (0.8679)	0.0028 (0.8638)
*State*	−0.2518*** (−6.3288)	−0.2519*** (−6.3314)
*ROA*	−0.4983*** (−2.9367)	−0.4961*** (−2.9266)
*TobinQ*	0.0244*** (2.7144)	0.0245*** (2.7158)
*Lev*	−0.0165 (−0.1727)	−0.0161 (−0.1688)
*Liquidity*	1.0003*** (10.8020)	1.0001*** (10.7989)
*Dual*	0.0211 (0.6473)	0.0211 (0.6463)
*Lshare*	−0.3025*** (−2.5845)	−0.3021*** (−2.5821)
*Indep*	0.4431* (1.6746)	0.4437* (1.6770)
*_cons*	−6.5089*** (−15.1567)	−6.5071*** (−15.1487)
Year	Yes	Yes
Industry	Yes	Yes
adj_*R*^2^	0.564	0.564
*N*	27,782	27,782

t statistics in parentheses; *p < 0.1, **p < 0.05, ***p < 0.01.

Second, the category of CEOs’ FB and shadow banking business of non-financial enterprises. To investigate the potential impact of CEOs’ FB categories on shadow banking business of non-financial enterprises, we estimated the parameters of the model shown in Equation 2. The regression results are shown in Column (2) of Table 3. *Bank_Fin* is positively correlated with *lnShadow*, but failed to pass the significance test. The coefficient of *NBank_Fin* is significantly positive at the level of 5%, indicating that CEOs with non-banking FBs are positively correlated with shadow banking business of non-financial enterprises. H2a is verified.

### Robustness Checks

We performed robustness tests by replacing the measurement methods, transforming the sample intervals, controlling for other shocks, changing the parameter estimation methods, and considering the endogeneity problem.

#### Replace the Measurement of Variables, Transform the Sample Interval, Control Other Factors, and Change the Parameter Estimation Method

##### Replace the Measurement of Variables

Alternative measurement for shadow banking business of non-financial enterprises. Referring to Han et al. (2019) and Li and Han (2019), we adopted the ratio of the sum of entrusted loans, entrusted finance, and private lending to total assets for measurement (*lnShadow1*), and the results are shown in Column (1) of Table 4. The coefficient of *CEO_Fin* is significantly positive at the 5% level, indicating that non-financial enterprises in which CEOs with FBs are more inclined to engage in shadow banking business and have larger shadow banks.

**TABLE 4:** Replace the measurement of the explained variable, transform the sample interval, control for other factors, and change the parameter estimation method.

	(1) Replace the measurement	(2) Transform the sample interval	(3) Control for other factors	(4) Bootstrap estimation method
*CEO_Fin*	0.0157** (2.3223)	0.1418** (2.3239)	0.1552** (2.5653)	0.1542** (3.6289)
*lnSize*	0.0059*** (3.7679)	1.1122*** (59.1285)	1.0925*** (59.4661)	1.0900*** (117.8207)
*Age*	0.0000 (0.1389)	0.0009 (0.2896)	0.0034 (1.0643)	0.0028* (1.6798)
*State*	−0.0206*** (−5.9639)	−0.2658*** (−6.5444)	−0.2429*** (−6.0567)	−0.2518*** (−12.4892)
*ROA*	−0.0335 (−1.5722)	−0.4033** (−2.3226)	−0.4816*** (−2.7746)	−0.4983*** (−3.8331)
*TobinQ*	0.0053*** (6.1147)	0.0194** (2.1009)	0.0242*** (2.6969)	0.0244*** (3.9598)
*Lev*	−0.1460*** (−17.8128)	−0.2312** (−2.3150)	−0.0279 (−0.2914)	−0.0165 (−0.2996)
*Liquidity*	0.1550*** (19.1519)	0.9676*** (10.1222)	0.9922*** (10.6990)	1.0003*** (20.3227)
*Dual*	0.0086*** (2.5855)	0.0216 (0.6480)	0.0422 (1.2411)	0.0211 (1.0124)
*Lshare*	0.0268*** (2.6346)	−0.2207* (−1.8460)	−0.3008** (−2.5626)	−0.3025*** (−4.7593)
*Indep*	0.0434 (1.6252)	0.3881 (1.4338)	0.4360* (1.6492)	0.4431*** (2.8770)
*CEO_Age*			−0.0052** (−2.4395)	
*CEO_Sex*			−0.0202 (−0.3110)	
*CEO_ZZ*			−0.0448 (−1.2951)	
*CEO_CW*			−0.0886* (−1.7926)	
*_cons*	−0.1075*** (−2.9837)	−7.2380*** (−16.0095)	−6.2832*** (−14.3259)	−6.5089*** (−30.5757)
Year	Yes	Yes	Yes	Yes
Industry	Yes	Yes	Yes	Yes
adj_*R*^2^	0.117	0.550	0.564	0.564
*N*	27,782	25,065	27,652	27,782

t statistics in parentheses; *p < 0.1, **p < 0.05, ***p < 0.01.

##### Transform the Sample Interval

In response to the global financial crisis of 2008, China implemented a series of rescue policies, such as the “four trillion” stimulus plan, which contributed to the growth of shadow banking business in a certain way. Therefore, to eliminate the impact of the 2008 financial crisis on our study and consider the possible lag of policy effects, we deleted the samples of 2008 and 2009 for re-estimation. The results are shown in Column (2) of Table 4. The coefficient of *CEO_Fin* is significantly positive at the 5% level, indicating that the results are robust.

##### Control for the Effects of Other Factors

Considering the impact of CEO heterogeneity on the shadow banking business of non-financial enterprises, we further controlled the effects of CEO age (*CEO_Age*), gender (*CEO_Sex*), political affiliation (*CEO_ZZ*), accounting background (*CEO_CW*), and other factors based on the baseline model. After controlling for CEO characteristics, the regression results are shown in Column (3) of Table 4. The results show that the relationship between CEOs’ FB and shadow banking business of non-financial enterprises remains unchanged.

##### Change the Parameter Estimation Method

To further eliminate the bias resulting from the self-selection of samples, we used the bootstrap self-sampling method for repeated sampling of the original samples. As shown in Column (4) of Table 4, the coefficient of *CEO_Fin* is significantly positive at the 5% level, indicating that the results are robust.

#### Endogeneity Problems

##### Lagged Variables

Given the possible endogeneity problem of reverse causation, we conducted a regression analysis on the CEO’s FB with a lag period, and the results are shown in Column (1) of Table 5. Additionally, to eliminate any interference from the control variables, we performed regression analysis using the control variables with a one-period lag. The results are shown in Column (2) of Table 5. The coefficient of *CEO_Fin* is significantly positive at the 5% level, indicating that the results are robust.

**TABLE 5:** Lagged variables, instrumental variables, and PSM parameter estimation.

	(1) Lagged explanatory variable	(2) Lagged control variable	(3) Instrumental variable	(4) PSM parameter estimation
*CEO_Fin*	0.1555** (2.5207)	0.1514** (2.4453)		0.1771** (2.4446)
*IV*			1.1554** (2.2084)	
*lnSize*	1.1066** (56.9458)	1.1042*** (55.9771)	1.0894*** (58.7678)	1.0365*** (25.9746)
*Age*	−0.0000 (−0.0003)	−0.0001 (−0.0297)	0.0021 (0.6560)	−0.0013 (−0.1792)
*State*	−0.2816*** (−6.7601)	−0.2899*** (−6.8440)	−0.2265*** (−5.3853)	−0.1797* (−1.7554)
*ROA*	−0.5573*** (−3.1323)	0.3084 (1.3679)	0.4634*** (−2.6920)	0.0667 (0.1731)
*TobinQ*	0.0198* (1.9459)	0.0547*** (5.4645)	0.0202** (2.1573)	−0.0143 (−0.7174)
*Lev*	−0.2362** (−2.2670)	−0.1587 (−1.5389)	−0.0294 (−0.3066)	0.2160 (1.0272)
*Liquidity*	1.1440*** (11.3288)	1.0230*** (10.2529)	1.0335*** (10.8932)	0.9564*** (4.4892)
*Dual*	0.0399 (1.1443)	0.0111 (0.3156)	−0.0057 (−0.1588)	0.0520 (0.6661)
*Lshare*	−0.2840** (−2.2690)	−0.2783** (−2.2444)	−0.2876** (−2.4390)	−0.0144 (−0.0522)
*Indep*	0.3174 (1.1325)	0.5268* (1.8557)	0.4177 (1.5710)	0.4992 (0.7796)
*_cons*	−7.0424*** (−15.0630)	−7.0030*** (−14.9297)	−6.5863*** (−15.1639)	−5.4576*** (−6.0314)
Year	Yes	Yes	Yes	Yes
Industry	Yes	Yes	Yes	Yes
adj_*R*^2^	0.555	0.538	0.555	0.558
*N*	23,435	23,435	27,782	2,449

t statistics in parentheses; *p < 0.1, **p < 0.05, ***p < 0.01.

##### Instrumental Variables

Given the potential endogeneity problems between CEOs’ FB and the shadow banking business of enterprises, we attempted to address it using the instrumental variable method. Referring to Faccio et al. (2006), we adopted the industry and year means of CEOs’ FB as instrumental variables (*IV*). The regression results of the second stage are shown in Column (3) of Table 5. The coefficient of *IV* is significantly positive at the 1% level, which is consistent with the result of the original model and can mitigate the potential endogeneity problems.

##### The Propensity Score Matching Method

To overcome the problem of sample selection bias, we screened the sample among the non-financial enterprises of CEOs without FBs that are more similar to the non-financial enterprises of CEOs with FBs in some financial indicators. After selecting the control group using PSM, we reran the regression using the new sample. The regression results are shown in Column (4) of Table 5. We can find that CEOs’ FB has a positive effect on shadow banking business of non-financial enterprises, which is consistent with the results of the original model, indicating that the results are robust.

## Mechanism Analysis

The above studies provide micro-level empirical support for a deeper understanding of the influencing factors of shadow banking business of non-financial enterprises. However, it is difficult to further clarify how CEOs’ FB affects the shadow banking business of non-financial enterprises by describing only the influence between “CEOs’ FB and shadow banking business of non-financial enterprises.” Therefore, it is necessary to open the mechanism black box and identify the path that the FB of the CEO affects the shadow banking business of non-financial enterprises. In this regard, we selected “the overconfidence and entity investment level of enterprises” to verify.

### CEOs’ Financial Background, Overconfidence, and Shadow Banking Business of Non-financial Enterprises

The upper echelon theory suggests that the background characteristics of managers may affect their psychometric factors, such as cognitive abilities, personal beliefs, and values, which, in turn, affect their behavior. In a psychological sense, overconfidence manifests itself overestimating of the odds of success and underestimating of the odds of failure (Wolosin et al., 1973). Psychological bias is prevalent among managers (Roll, 1986; Landier and Thesmar, 2009), causing them to overestimate their capabilities and the returns of investment projects, but underestimating the risk exposure involved with those projects. CEOs with FBs have a competitive advantage over CEOs without a FB in understanding investments, controlling risks, grasping opportunities, and processing information. Due to their greater business experience and stronger professional qualifications in the field of investment, they believe that they are more likely to succeed in investment projects and create greater value for enterprises (Cheng and Wu, 2022). Therefore, CEOs with FBs may be overconfident, while overconfident CEOs prefer high-risk shadow banking businesses, such as entrusted loans (Wu et al., 2021). Thus, we attempted to use overconfidence (*Overcon*) as a mediator variable. Referring to Hayward and Hambrick (1997); Liu and Li (2021), et al., we employed the executive relative compensation method to measure managerial overconfidence (*Overcon*). Specifically, we compared the ratio of the sum of the top three executives’ compensation and the sum of all the top executives’ compensation with the median. If the ratio is greater than the median, the value is 1; otherwise, it is 0.

Table 6 shows the estimation results for overconfidence as a mediating variable. It can be seen from Table 6 that the coefficient of the explanatory variable (*CEO_Fin*) is significantly positive at the 5% level in Column (1). In Column (2), the coefficients of the explanatory variable (*CEO_Fin*) and mediating variable (*Overcon*) are significantly positive at the 5% level, indicating that CEOs with FBs develop overconfidence. In Column (3), we included both the explanatory variable (*CEO_Fin*) and the mediating variable (*Overcon*), and the coefficient between the mediating variable (*Overcon*) and the explained variable (*lnShadow*) was not significant. Furthermore, we conducted the Sobel test, and *Z*-statistic was 0.8469 and *p*-value was 0.3970, which failed the significance test. Therefore, overconfidence is not a channel for CEOs with FBs to influence the shadow banking business of non-financial enterprises. This is different from the findings of Du et al. (2019), who argue that CEOs’ FB promotes enterprises financialization by increasing CEOs’ confidence. This also confirms from the side that, although shadow banking business of non-financial enterprises still belongs to the category of enterprise financialization in essence, it is distinct from investing in mainstream financial assets, such as stocks and bonds.

**TABLE 6:** CEOs’ FB, overconfidence, and shadow banking business of non-financial enterprises.

	(1) *lnShadow*	(2) *Overcon*	(3) *lnShadow*
*CEO_Fin*	0.1542** (2.5416)	0.0556** (2.5357)	0.1533** (2.5287)
*Overcon*			0.0150 (0.5554)
*lnSize*	1.0900*** (59.3066)	−0.0649*** (−9.6706)	1.0910*** (59.3205)
*Age*	0.0028 (0.8679)	0.0060*** (4.6320)	0.0027 (0.8398)
*State*	−0.2518** (−6.3288)	−0.1212*** (−7.6425)	−0.2500*** (−6.2644)
*ROA*	−0.4983*** (−2.9367)	−0.0055 (−0.0964)	−0.4982*** (−2.9354)
*TobinQ*	0.0244*** (2.7144)	0.0123*** (4.4465)	0.0243*** (2.6865)
*Lev*	−0.0165 (−0.1727)	−0.0215 (−0.6953)	−0.0161 (−0.1692)
*Liquidity*	1.0003*** (10.8020)	−0.0760** (−2.3204)	1.0015*** (10.8201)
*Dual*	0.0211 (0.6473)	0.0280** (2.2401)	0.0207 (0.6343)
*Lshare*	−0.3025*** (−2.5845)	0.2127*** (4.7602)	−0.3057*** (−2.6053)
*Indep*	0.4431* (1.6746)	0.5965*** (5.8062)	0.4342 (1.6345)
*_cons*	−6.5089*** (−15.1567)	1.5940*** (10.3336)	−6.5328*** (−15.2191)
Year	Yes	Yes	Yes
Industry	Yes	Yes	Yes
adj_*R*^2^	0.564	0.061	0.564
*N*	27,782	27,782	27,782

t statistics in parentheses; *p < 0.1, **p < 0.05, ***p < 0.01.

### CEOs’ Financial Background, Entity Investment Level, and Shadow Banking Business of Non-financial Enterprises

Enterprises’ resources are often limited, as are their financial resources. Demir (2009) found that financial investment by non-financial enterprises will crowd out investment in the real economy. If enterprises spend too much money on shadow banking activities, they will not be able to upgrade their facilities and develop new products. As enterprises’ profits from investment in the real industry keep decreasing, while those from financial assets keep increasing, enterprises reduce their investment in fixed assets (Tori and Onaran, 2018). In particular, enterprises with CEOs with FBs have even less endogenous incentives to make real investments (Liu et al., 2020), and they prefer to invest in the shadow banking business with funds originally used for their main business or other businesses. CEOs with FBs impact shadow banking business of non-financial enterprises by influencing enterprises’ willingness to invest in real entities. Therefore, we examined enterprise entities investment levels (*Entity*) as a mediating variable. Referring to Han and Li (2020), we adopted the natural logarithm of the cash paid for the purchase of fixed assets, intangible assets, and other long-term assets to measure it.

Table 7 shows the estimated results for the level of entity investment as a mediating variable. As shown in Table 6, the estimated coefficient of the explanatory variable (*CEO_Fin*) in Column (1) is significantly positive at the 5% level. The coefficient of the explanatory variable (*CEO_Fin*) and the mediating variable (*Entity*) in Column (2) is significantly negative at the 1% level, indicating that CEOs with FBs will reduce the level of enterprise entities investment. In Column (3), we included both the explanatory variable (*CEO_Fin*) and the mediating variable (*Entity*), and the coefficients of the mediating variable (*Entity*) and the explained variable (*CEO_Fin*) are significantly positive. Furthermore, we conducted the Sobel test and found that the *Z*-statistic was −6.101 and significant at the 1% level, indicating that the level of entity investment partially mediates the relationship between the CEO’s FB and the shadow banking business of non-financial enterprises. CEOs with FBs influence the shadow banking business of non-financial enterprises by reducing the level of entity investment. As a result, the findings support the path of “CEO’s FB → enterprises’ entity investment levels → shadow banking business of non-financial enterprises.”

**TABLE 7:** CEOs’ FB, entity investment level, and shadow banking business of non-financial enterprises.

	(1) *lnShadow*	(2) *Entity*	(3) *lnShadow*
*CEO_Fin*	0.1542** (2.5416)	−0.2436*** (−4.4480)	0.1728*** (2.8379)
*Entity*			0.0767*** (5.6673)
*lnSize*	1.0900*** (59.3066)	1.0906*** (77.4214)	1.0064*** (42.0365)
*Age*	0.0028 (0.8679)	−0.0359*** (−12.7190)	0.0056* (1.7275)
*State*	−0.2518*** (−6.3288)	−0.1979*** (−6.1095)	−0.2366*** (−5.9624)
*ROA*	−0.4983*** (−2.9367)	2.7078*** (16.9303)	−0.7059*** (−4.1429)
*TobinQ*	0.0244*** (2.7144)	−0.0103 (−1.3526)	0.0252*** (2.8411)
*Lev*	−0.0165 (−0.1727)	−0.3534*** (−3.7343)	0.0106 (0.1112)
*Liquidity*	1.0003*** (10.8020)	−1.8186*** (−19.8392)	1.1398*** (11.9430)
*Dual*	0.0211 (0.6473)	0.1301*** (5.1777)	0.0112 (0.3420)
*Lshare*	−0.3025*** (−2.5845)	0.2041** (2.1737)	−0.3181*** (−2.7216)
*Indep*	0.4431* (1.6746)	−0.3875* (−1.7316)	0.4729* (1.7875)
*_cons*	−6.5089*** (−15.1567)	−3.3385*** (−10.185)	−6.2529*** (−14.5137)
Year	Yes	Yes	Yes
Industry	Yes	Yes	Yes
adj_*R*^2^	0.564	0.653	0.566
*N*	27,782	27,782	27,782

t statistics in parentheses; *p < 0.1, **p < 0.05, ***p < 0.01.

## Extended Analysis

Our study investigates whether there are significant differences in the positive effects of CEOs’ FBs on shadow banking business of non-financial enterprises, depending on different factors, such as internal micro characteristics and external macro factors. Micro characteristics of enterprises are analyzed in terms of equity nature, industry nature, and life cycle; macro characteristics of enterprises are analyzed in monetary policy, industry competition, and institutional environment.

### Internal Micro Characteristics of Enterprises

#### The Heterogeneity of Nature of Equity

Drawing on existing studies, we further divided the samples into state-owned enterprises (SOEs) and non-SOEs, taking 1 and 0, respectively, and the estimated results are shown in Table 8. From Columns (1) and (2) of Table 8, it can be seen that CEOs’ FB is significantly and positively correlated with shadow banking business of SOEs at the 5% level. Although non-SOEs are positively correlated, they fail to pass the significance test. Non-SOEs in China continue to be at a disadvantage in allocating credit resources due to the phenomenon of “ownership discrimination” (Allen et al., 2005; Allen et al., 2019). SOEs are more likely to receive government subsidies and financial support than non-SOEs (Wang et al., 2021). CEOs of SOEs with FBs are more likely to engage in the shadow banking business, and the scale of shadow banking keeps increasing.

**TABLE 8:** CEOs’ FB and shadow banking business of non-financial enterprises: internal micro characteristics of enterprises.

	(1) SOEs	(2) Non-SOEs	(3) MEs	(4) Non-MEs	(5) Growing enterprise	(6) Non-growth enterprise
*CEO_Fin*	0.2901** (2.4508)	0.1146 (1.5965)	0.0477 (0.5577)	0.2671*** (3.1766)	0.1016 (1.3526)	0.1784** (2.4007)
*lnSize*	1.1102*** (36.6023)	1.1158*** (50.8724)	1.0796** (42.7222)	1.1299*** (44.0496)	1.1134** (59.7001)	1.0754*** (43.1434)
*Age*	0.0014 (0.2261)	0.0035 (0.9348)	0.0015 (0.3636)	0.0058 (1.0887)	0.0060* (1.7343)	−0.0019 (−0.4507)
*State*			−0.2858*** (−5.6932)	−0.1625** (−2.4730)	−0.1763*** (−4.0574)	−0.3201*** (−6.3799)
*ROA*	−1.1033*** (−2.7744)	−0.4916*** (−2.6759)	−0.5723** (−2.5207)	−0.5868** (−2.3354)	−0.3430 (−1.3187)	−0.5025*** (−2.5933)
*TobinQ*	0.0320 (1.5620)	0.0216** (2.2368)	0.0311** (2.5635)	0.0180 (1.4296)	0.0435*** (4.2803)	0.0091 (0.7420)
*Lev*	0.1584 (0.9585)	−0.2442** (−2.1069)	−0.0666 (−0.5460)	0.1425 (0.9101)	0.0116 (0.1110)	−0.0360 (−0.2994)
*Liquidity*	1.2185*** (7.7315)	0.9254*** (8.5352)	1.0525*** (8.5592)	0.9910*** (7.1624)	0.6756*** (6.7123)	1.2855*** (10.6344)
*Dual*	0.0460 (0.6324)	−0.0063 (−0.1738)	0.0759* (1.9380)	−0.1103* (−1.8888)	−0.0234 (−0.6389)	0.0776* (1.8394)
*Lshare*	−1.0496*** (−5.1656)	0.2184 (1.6020)	−0.2672* (−1.8452)	−0.4452** (−2.2128)	−0.1773 (−1.4173)	−0.4059*** (−2.7372)
*Indep*	0.4777 (1.1599)	0.2707 (0.8180)	0.4896 (1.4500)	0.3427 (0.8287)	0.6400** (2.1532)	0.2492 (0.7386)
*_cons*	−6.5395*** (−9.5549)	−7.4717*** (−14.4009)	−6.7498*** (−11.6693)	−7.2448*** (−12.9081)	−7.4265** (−17.0351)	−5.8539*** (−10.0452)
Year	Yes	Yes	Yes	Yes	Yes	Yes
Industry	Yes	Yes	Yes	Yes	Yes	Yes
adj_*R*^2^	0.628	0.535	0.536	0.617	0.605	0.536
*N*	10,547	17,235	17,995	9,787	12,976	14,806

t statistics in parentheses; *p < 0.1, **p < 0.05, ***p < 0.01.

#### The Heterogeneity of Industry Nature

Based on existing studies, we further divided the samples into manufacturing enterprises (MEs) and non-MEs, taking 1 and 0, respectively. The estimated results are shown in Table 8. As can be seen from Columns (3) and (4) of Table 8, the FB of the CEO is significantly and positively correlated with shadow banking business of non-MEs at the 1% level. Although the two are positively correlated, they fail to pass the significance test for MEs. According to the findings, CEOs with a FB are more likely to engage in shadow banking activities in non-MEs. There may be a serious economic downturn present, explaining this phenomenon. As the core of China’s real economy, MEs are responsible for promoting high-quality economic growth and the development of innovative transformation. MEs are facing very severe survival conditions.

#### The Heterogeneous of Life Cycle

Referring to Dickinson (2011), we chose the cash flow combination to divide enterprises into growing and non-growth enterprises. As can be seen from Columns (5) and (6) of Table 8, the FB of the CEO is significantly and positively correlated with shadow banking business of non-growth enterprises at the 5% level. Although the two are positively correlated for enterprises in the growth stage, they fail to pass the significance test. The study indicates that CEOs with a FB are more likely to engage in shadow banking activities within enterprises that are not in growth stages. A possible reason for this result is that enterprises in non-growth stages typically have more resources than those in growth stages.

### External Macro Environment of Enterprise

#### The Heterogeneous of Monetary Policy

Referring to Dai and Yue (2015), we used the difference between the growth rate of nominal GDP and the growth rate of money (M1) and quasi-money (M2) supply to measure the easing and tightening of monetary policy. If the difference between the two is greater than the median, the value is 1, indicating monetary policy tightening. Otherwise, the value is 0. The estimated results are shown in Table 9. As can be seen from the estimated coefficients in Columns (1) and (2) of Table 9, the FB of CEOs is significantly and positively related to the shadow banking business of non-financial enterprises at the 10% level, regardless of whether monetary policies are in a period of easing or tightening. However, the positive effect is greater in the period of easing monetary policy. Excess money issuance is an important inducement for enterprises to engage in shadow banking business (Yang et al., 2019). In the period of easing monetary policy, the financing environment of non-financial enterprises is relatively loose, and CEOs do not need to be anxious about the availability of capital liquidity in the future. An excessive supply of liquidity at the macro level induces money to flow into the financial market, generating high profit returns and causing extremely active financial market transactions. At this time, the companies of CEOs with FBs are more inclined to engage in shadow banking business.

**TABLE 9:** CEOs’ FB and shadow banking business of non-financial enterprises: external macro characteristics of enterprises.

	(1) Tight monetary policy	(2) Easy monetary policy	(3) Higher industry competition	(4) Lower industry competition	(5) Better institutional environment	(6) Worse institutional environment
*CEO_Fin*	0.1265* (1.8056)	0.1871** (2.2501)	0.1693** (2.0027)	0.1403 (1.6237)	0.1669** (2.0289)	0.1235 (1.4375)
*lnSize*	1.0458*** (58.1581)	1.1474*** (47.5250)	1.1005*** (44.1818)	1.0910*** (41.7996)	1.1451*** (51.8232)	1.0652*** (40.8429)
*Age*	0.0034 (1.0377)	0.0024 (0.5581)	0.0038 (0.8151)	0.0015 (0.3350)	−0.0074* (−1.8615)	0.0148*** (3.0834)
*State*	−0.1972*** (−4.9115)	−0.3062*** (−6.1414)	−0.1857*** (−3.3865)	−0.2908** (−5.0549)	−0.1995*** (−3.3349)	−0.2787*** (−5.5701)
*ROA*	−0.4664*** (−2.9459)	−0.4243 (−1.0487)	−0.7022*** (−2.8660)	−0.3898* (−1.6584)	−0.4677** (−2.1859)	−0.7539*** (−3.0174)
*TobinQ*	0.0371*** (2.9933)	0.0238** (2.3013)	0.0402*** (2.8849)	0.0103 (0.9055)	0.0150 (1.3187)	0.0261* (1.8753)
*Lev*	0.1261 (1.3381)	−0.1653 (−1.2715)	−0.0200 (−0.1454)	−0.0388 (−0.2922)	−0.7002*** (−5.3091)	0.4268*** (3.4632)
*Liquidity*	0.9704*** (10.2935)	1.0203*** (8.4727)	1.0258*** (8.1088)	0.9485*** (7.2066)	0.9609*** (7.9387)	1.1192*** (8.7101)
*Dual*	0.0276 (0.7855)	0.0156 (0.3405)	0.0706 (1.6048)	−0.0402 (−0.8239)	−0.0072 (−0.1723)	0.0052 (0.1086)
*Lshare*	−0.3861*** (−3.1993)	−0.2498* (−1.6594)	−0.1334 (−0.8262)	−0.3925** (−2.3042)	0.0730 (0.5011)	−0.6378*** (−3.7574)
*Indep*	0.6695** (2.3226)	0.1653 (0.4816)	0.1327 (0.3656)	0.7351* (1.9342)	0.3329 (0.9970)	0.4650 (1.2104)
*_cons*	−5.7042*** (−13.2685)	−7.7931*** (−14.0936)	−6.8119*** (−12.6439)	−6.5774*** (−10.9684)	−8.1344*** (−14.6974)	−6.2028*** (−10.1676)
Year	Yes	Yes	Yes	Yes	Yes	Yes
Industry	Yes	Yes	Yes	Yes	Yes	Yes
adj_*R*^2^	0.606	0.518	0.553	0.576	0.539	0.577
*N*	14,716	13,066	13,789	13,993	13,824	13,958

t statistics in parentheses; *p < 0.1, **p < 0.05, ***p < 0.01.

#### The Heterogeneity of Market Competition

According to Nickell (1996), we adopted the standard deviation of the profit margin of the industry’s main business to measure industry competition. The smaller the value, the more competitive the industry will be. If the standard deviation of the industry’s main business profit margin is less than the median, the value is 1, indicating higher industry competition. Otherwise, the value is 0, indicating lower industry competition. The estimated results are shown in Table 9. As can be seen from Columns (3) and (4) of Table 9, in industries with higher competition, the FB of CEOs is significantly and positively correlated with shadow banking business of non-financial enterprises at the 5% level. Although the two are positively correlated in the industry with a lack of competition, they fail to pass the significance test. The study indicates that the positive effect is more significant when the industry is more competitive. Enterprises in higher competitive industries may have more urgent financing needs to expand new business areas and reduce operating risks. At the same time, market competition also forces enterprises to minimize the information asymmetry between the supply and demand of funds so that external capital can be acquired at a lower cost (Yi et al., 2010). CEOs with FBs are more inclined to leverage their financial expertise to engage in shadow banking activities at this time.

#### The Heterogeneity of the Institutional Environment

According to the Report on Marketization Index by Provinces in China (2018) by Fan and Wang, if the marketization index is greater than the median, the value is 1, indicating a better institutional environment. Otherwise, the value is 0, indicating a worse institutional environment. The estimated results are shown in Table 9. As can be seen from Columns (5) and (6) of Table 9, the FB of the CEO is significantly and positively correlated with the shadow banking business of non-financial enterprises at the 5% level when the institutional environment is better. While it does not pass the significance test when the institutional environment is worse, although they are positively correlated. Shadow banking grows in scale with the improvement of economic growth (Tan et al., 2021). Shadow banking is a product of financial development at a certain stage. The phenomenon tends to occur in areas with a relatively high degree of marketization and relatively diversified capital. When the institutional environment is better, there is relatively less government intervention in enterprises, and CEOs have higher decision-making discretion (Wei and Ling, 2015). There is a more relaxed investment environment at this time, and CEOs with FBs can leverage their financial advantages to engage in shadow banking business.

## Conclusion and Implications

### Main Conclusion

The “finance-like” behavior of non-financial enterprises has encouraged the “off real to virtual,” which has seriously restricted the virtuous cycle of finance and economy. In the long run, to explore the long-term mechanism of shadow banking governance, we need to focus on its root, guide non-financial enterprises to carry out shadow banking business in a reasonable and compliant manner, and promote the improvement of China’s financial market and the high-quality economic development. This study analyzes samples of non-financial enterprises in China’s A-share-listed companies from 2008 to 2019 and empirically tests the influence and mechanism of the CEO’s FB on the shadow banking business of non-financial enterprises. The results show that, first, the CEO’s FB positively affects the shadow banking business of non-financial enterprises. Among them, the positive effect of non-bank FB is stronger. The results are still valid after robustness tests by replacing the measurement of variables, transforming the sample interval, controlling for other shocks, changing the parameter estimation method, and considering the endogeneity problem. Second, the mechanism analysis shows that CEOs’ FB mainly promotes the shadow banking business of non-financial enterprises by reducing investment in corporate entities. Third, the heterogeneity analysis finds that the relationship between the two is affected by different situational factors, such as the internal and external environments of enterprises. On the one hand, in terms of the micro characteristics of enterprises, the positive effect is more obvious in SOEs, non-MEs, and non-growth enterprises. On the other hand, with respect to the external macro environment, CEOs with FBs have a stronger positive impact on the shadow banking business of non-financial enterprises in the period of easy monetary policy, in industries with higher competition or regions with better institutional environment. Our study reveals the internal mechanism of the CEO’s FB and shadow banking business of non-financial enterprises. This study also enriches the research into the influencing factors of shadow banking business of non-financial enterprises and provides micro-level empirical support to alleviate the “off real to virtual” of the economy.

### Implications

From the perspective of the government, first, the government should effectively reduce the burden and operating cost of enterprises, promote the transformation and upgrading of enterprises, enhance the core competitiveness of enterprises in the real economy, and improve the benefits of investment in the real economy. Second, the government can strengthen the “penetrating supervision” of the financial system, integrate the shadow banking market into the formal market, break through the boundary between formal and informal finances, and improve the structural regulation function of financial services in the real economy. Third, the government can take advantage of data resources to promote the integration of digitalization and marketization, establish a multilevel financial market, and create a favorable market environment for non-financial enterprises.

From the perspective of enterprises, first, enterprises need to establish a mechanism for evaluating management experience and explore the potential advantages of utilizing the previous experience of managers. In addition, when selecting the management team, the enterprise should also consider the different backgrounds of members, fully understand how the team’s members complement one another, and encourage the members to perform their managerial duties effectively. Second, enterprises should develop a compensation system linked to the cost of risk, establish and improve the relevant performance assessment mechanism, and restrain the excessive speculative behavior of its decision makers. Third, enterprises can use big data to conduct in-depth research and explore market demand, improve the enthusiasm of non-financial enterprises to carry out their main businesses, and increase the expected benefits of industrial investment.

In addition, senior management should grasp the current situation of the enterprise and avoid habitual thinking. The senior management should, depending on the actual situation of the enterprise, clarify the extent of risks it can take to prevent the enterprise from hindering or even damaging the development of its main business.

## Data Availability Statement

The raw data supporting the conclusions of this article will be made available by the authors, without undue reservation.

## Author Contributions

CY: research design, data collection, sorting, analysis, and drafting the manuscript. WS: modifying the manuscript. Both authors contributed to the article and approved the submitted version.

## Conflict of Interest

The authors declare that the research was conducted in the absence of any commercial or financial relationships that could be construed as a potential conflict of interest.

## Publisher’s Note

All claims expressed in this article are solely those of the authors and do not necessarily represent those of their affiliated organizations, or those of the publisher, the editors and the reviewers. Any product that may be evaluated in this article, or claim that may be made by its manufacturer, is not guaranteed or endorsed by the publisher.

## Footnotes

1 The data come from the “China’s Shadow Banking Report” released by the China Banking and Insurance Regulatory Commission.

2 The sum of the means of CEOs with banking background (*Bank_Fin*) and those with non-banking financial background (*NBank_Fin*) is less than that of CEOs with financial background (*CEO_Fin*). To better clarify the difference in the impact of the two on the shadow banking business of non-financial enterprises, we excluded CEOs who have both.
